# 

**Published:** 1974-10

**Authors:** 


The
Bristol Medico-Chirurgical Journal
(Incorporating the Medical Journal of the South-West)
Established 1883

Vol. 89 (iv) OCTOBER 1974 No. 332

### BRISTOL MEDICO-CHIRURGICAL JOURNAL

Published Quarterly

Annual Subscription £2 Post Free

### BRISTOL MEDICO-CHIRURGICAL SOCIETY

**President 1973-74: Dr. R. F. Barbour**

**President-elect: Dr. J. Apley**

**Honorary Secretary: Dr. I. S. Bailey, 7 Percival Road, Bristol 8.**

**Honorary Treasurer: Dr. Frank Ross, Bristol Royal Infirmary, Bristol 2.**

The Society was founded in 1874. In 1883 publication of the Journal began and has continued quarterly ever since then. During the years 1949 to 1962 it appeared under the title of "The Medical Journal of the South West".

### THE BRISTOL MEDICO-CHIRURGICAL JOURNAL

**Editorial Committee**

Editor Dr. N. J. Brown, Southmead Hospital, Bristol.

Assistant Editor Dr. D. S. Reeves.

Members Dr. Rhys Davies.
Dr. M. B. Lennard.
Professor R. C. Wofinden.
Dr. D. W. Wright.
The Hon. Treasurer and Hon. Secretary of the Society.

Business Manager Dr. P. J. F. Baskett, Frenchay Hospital, Bristol.

Secretary Mr. B. P. Jones, The Library, Medical School, University Walk, Bristol BS8 1TD.

### NOTICE TO CONTRIBUTORS

The Journal is especially concerned to provide a place for the recording of medical thought, interests, and practice in and around Bristol. It therefore welcomes articles that, as well as being of immediate interest to members, will document the local and contemporary medical sc ene for our successors.

Original articles are invited provided that they have not been published elsewhere. References in the text should be by author's name, with year of publication in parenthesis; they should be listed at the end in alphabetical order, the name of each journal or book being given in full—not abbreviated—and the title of the article added. Illustrations should be supplied ready for reproduction. Line drawings should be in Indian ink on firm white paper or board. The author will be informed if it is found necessary to ask him to pay for the making of blocks.

The following contributions will be especially welcome; notes of forthcoming events, meetings held, degrees conferred, staff appointments, honours and public appointments and other local news.

### The Medical Library

In 1890 the Bristol Medico-Chirurgical Society founded a medical library which in 1892 was combined with that of the Bristol Medical School. This became the University of Bristol Medical Library in 1925 and an agreement in that year confirmed the privilege of Members of the Society to use the University Medical Library.

### A Selection of Recent Additions to the Medical Library

LANGMAN, M. J. S.: A concise textbook of gastroenterology. (Churchill Livingstone)

LEE, D. H. K.: Metallic contaminants & human health. (Acad. Press)

LEPOW, I. H. & P. A. WARD eds.: Inflammation: mechanisms and control. (Acad. Press)

LITTLE, K.: Bone behaviour. (Acad. Press)

LONGO, V. G.: Neuropharmacology of behaviour. (Freeman)

MARSHALL, C. L. & D. PEARSON: Dynamics of health and disease. (Appleton-Century-Crofts)

MENDELS, J. & S. K. SECUNDA: Lithium in medicine. (Gordon & Breach)

MENDELSOHN, E. et al eds.: Human aspects of biomedical innovation. (Harvard Univ. Press)

MILLER, J. B.: Food allergy: provocative testing & injection therapy. (Thomas)

MITTMAN, C. ed.: Pulmonary emphysema & proteolysis. (Acad. Press)

MODELL, J. H.: The pathophysiology & treatment of drowning & near-drowning. (Thomas)

MUIR, D. C. F.: Clinical aspects of inhaled particles. (Heinemann)

NAIR, P. P. & D. KRITCHEVSKY eds.: The bile acids: chemistry, physiology & metabolism. 2 vols. (Plenum Press)

NOLAND, R. L. ed.: Counselling parents of the emotionally disabled child. (Thomas)

NORDIN, B. E. C.: Metabolic bone & stone disease. (Churchill Livingstone)

NOUR-ELDIN, F.: Haematology: rudimental, practical & clinical. (Butterworths)

ORME, J. E. & F. G. SPEAR: Psychology in medicine. (Baillière)

PETRILLO, M. & S. SANGER: Emotional care of hospitalized children: an environmental approach. (Lippincott)

PHILLIPS, E. D.: Greek medicine. (Thames & Hudson)

PITNEY, W. R.: Clinical aspects of thromboembolism. (Churchill Livingstone)

PLANTEVIN, O. M.: Analgesia and anaesthesia in obstetrics. (Butterworths)

POLLACK, H. M.: Radiologic examination of the urinary tract. (Harper & Row)

POLLEN, A. G.: Fractures and dislocations in children. (Churchill Livingstone) Presented by Mr. D. M. Tanner.

RAIMONDI, A. J.: Pediatric neuroradiology. (Saunders)

RICHARDS, V.: Cancer—the wayward cell: its origins, nature and treatment. (California Univ. Press)

ROBERTS, D. F. & E. SUNDERLAND: Genetic variation in Britain. (Taylor & Francis)

ROSS, J. A. et al.: Behaviour of the human ureter in health and disease. (Churchill Livingstone)

SCHEIN, C. J.: Acute cholecystitis. (Harper & Row)

SCHILLING, R. S. F. ed.: Occupational health practice. (Butterworths)

SCHOENBERG, D. et al eds.: Psychosocial aspects of terminal care. (Columbia Univ. Press)

SELLINK, J. L.: Examination of the small intestine by means of duodenal intubation. (Stenfert Kroese)

SHIVAS, A. A. & S. C. FRASER: Frozen sections in surgical diagnosis. (Churchill Livingstone)

SMITH, C. A.: The critically ill child: diagnosis and management. (Saunders)

SMITH, B. J.: Fundamentals of anesthesia care. (Mosby)

SNAPPER, I. & A. I. KAHN: Myelomatosis: fundamentals and clinical features. (University Park Press)

SODEE, D. B. & P. J. EARLY: Technology and interpretation of nuclear medicine procedures. (Mosby)

SOLOMON, L. M. & N. B. ESTERLY: Neonatal dermatology. (Saunders)

SPELLER, S. R.: Law of doctor and patient. (Lewis)

TACHDJIAN, M. O.: Paediatric orthopedics. 2 vols. (Saunders)

TAYLOR, J. L.: The doctor and the law. (Pitman)

TONER, P. G. et al.: The digestive system: an ultrastructural atlas and review. (Butterworths)

VAN DER REIS, L. & H. P. LAZAR: The human digestive system: its functions and disorders. (Karger)

WARREN SPRINGS LABORATORY: National survey of air pollution, 1961-71. Vol. 2: South West Region, Wales Region, North West Region. (HMSO)

WHEATLEY, D.: Psychopharmacology in family practice. (Heinemann)

WIRZ, H. & F. SPINNELLI eds.: Recent advances in renal physiology. (Karger)

WISSLER, R. W. & J. C. GEER eds.: The pathogenesis of atherosclerosis. (Williams & Wilkins)

WOLF, P. L. et al.: Methods and techniques in clinical chemistry. (Wiley)

ZIPKIN, I. ed.: Biological mineralization. (Wiley)

### UNIVERSITY OF BRISTOL

### Degrees and Diplomas Awarded

**M.D.**
J. F. MORRIS
C. A. PENNOCK
H. STEINER

**M.B., Ch.B. with Honours**
J. T. BOURNE—with Distinctions in Medicine, Surgery, Child Health, Mental Health, Clinical Pathology, and in Community Medicine
Jennifer M. CADDY—with Distinction in Obstetrics and Gynaecology
J. R. THORNTON—with Distinction in Mental Health
Kathleen L. WELCHEW—with Distinction in Clinical Pathology

**M.B., Ch.B.**
Teresa M. ALLEN
P. G. McD. ANDER
R. S. ANDERSON—with Distinction in Child Health
C. W. ANDREWS
C. G. BATES
D. G. J. BATTIN
J. M. BIRD
Jennifer L. BOLT
J. P. G. BOLTON
Barbara E. BRADBURN
M. D. BREWIN
F. M. CANTOR
N. D. CARR
Susan M. CHALLEN
J. C. COGHILL
Kathryn E. COLLINGHAM
P. H. COOPER
R. J. COPPEN—with Distinction in Clinical Pathology
Joan F. CRAWFORD
D. R. CREAK
A. DAVIS
K. M. DE COCK
Margaret DIXON
Rosemary A. DODDINGTON
Rosemary A. DOVE
Janet P. DOWNER
D. J. EDEN
J. J. A. ELGON
J. A. ELLIOTT
T. C. EWER—with Distinctions in Child Health, Mental Health, and in Clinical Pathology
P. J. FINAN
G. P. C. FOSBROOKE
P. F. GODFREY
Patricia M. GOMERSALL
M. A. GREENSTONE
D. N. F. HARRIS
M. C. J. HELLIWELL
Susan J. HEROD
C. P. HINTON
J. H. HOBBISS
Cynthia M. HONEY
D. HONEYBOURNE
J. C. HOPKINS
Christine A. E. HOYTE
S. W. HUDSON
M. F. JOHNSON
Helen C. JONES—with Distinction in Clinical Pathology
Kathryn M. JONES
Madeleine J. KEYWORTH
Dorcas KINGHAM—with Distinction in Surgery
P. D. LATHAM
I. J. LEWIS
Irene J. LIGHTFOOT
K. B. LIM
P. S. LUTTER
D. D. R. MACAULAY
Claire M. MASCARENHAS
C. I. MORWOOD
J. A. MURRAY
Nichola J. OWEN
G. S. PAYNE
P. J. PHILLIPS
C. P. POLLAND
I. P. PRESCOTT
C. J. PRITCHETT
Linda S. PRYCE—with Distinction in Child Health
M. I. RICHARDSON
Marie-Christine RIMMER
M. SHEEN
R. J. STYLES—with Distinction in Child Health
Fay J. SUTTON
R. M. D. TRANTER
Susan M. TUCK
A. B. TULLO
Heather M. TURNER
Verity J. TYRRELL
A. J. W. VERNIQUET
Jacqueline V. WALSH
M. R. WATTS
Jane R. WEBSTER
E. A. WELCHEW
Margaret F. WILKINS
M. WILLIAMS
M. T. WYATT

**D.P.H.**
A. B. CHRISTIAN
V. KATSOUJIANNOPOULOS
K. P. LOUIS
A. MOORE
D. A. MOORE
R. PHILIP
R. PLATT
E. W. POWELL
T. S. RUBERU
C. SOMASUNDARAM
W. G. WESTALL

### INDEX TO VOLUME 89

| AUTHORS | Page |
|---|---|
| Barbour, R. F. | 45 |
| Barros, d'Sa, A. A. J. | 71 |
| Brown, N. J. | 31 |
| Cowie, Valerie A. | 69 |
| Jancar, J. | 1 |
| McPherson, A. G. | 11 |
| Perry, C. B. | 25 |
| Radford, P | 7 |
| Rees, J. D. | 5 |
| Roddie, R. K. | 17 |
| Webb, A. J. | 59 |

### SUBJECTS

| | |
|---|---|
| Angiofibroma, nasopharyngeal (Roddie, R. K.) ... ... ... ... ... ... | 17 |
| Biopsy, Needle aspiration (Webb, A. J.) ... ... ... ... ... ... ... | 59 |
| Bristol, History of medicine in (Perry, C. B.) ... ... ... ... ... ... | 25 |
| Bristol, History of psychiatry in (Barbour, R. F.) ... ... ... ... ... ... | 45 |
| Bristol Medico-Chirurgical Society ... ... ... ... ... ... ... | 10, 22, 55, 56, 58 |
| Bristol Medico-Chirurgical Society, History of (Perry, C. B.) ... ... ... ... | 25 |
| Gethin Shilling (Jancar, J.) ... ... ... ... ... ... ... ... ... | 1 |
| Journalism, Medical, in West of England (Brown, N. J.) ... ... ... ... ... | 31 |
| Medical Library ... ... ... ... ... ... ... ... ... ... | 9, 16, 76 |
| Medicine in Bristol (Perry, C. B.) ... ... ... ... ... ... ... ... | 25 |
| Mentally Handicapped, Dental surgical unit for (Rees, J. D.) ... ... ... ... | 5 |
| Mentally Handicapped, Care of (Cowie, Valerie A.) ... ... ... ... ... | 69 |
| Midwifery and the Egg (Radford, P.) ... ... ... ... ... ... ... | 7 |
| Needle aspiration biopsy (Webb, A. J.) ... ... ... ... ... ... ... | 59 |
| Newborn, Surgical problems (McPherson, A. G.) ... ... ... ... ... ... | 11 |
| News and Notes ... ... ... ... ... ... ... ... ... ... | 10, 22, 58, 77 |
| Pancreas, Solitary cyst (Barros, d'Sa A. A. J.) ... ... ... ... ... ... | 71 |
| Psychiatry in Bristol, History (Barbour, R. F.) ... ... ... ... ... ... | 45 |
| South West Orthopaedic Club ... ... ... ... ... ... ... ... | 75 |

